# Application of metal-organic frameworks in infectious wound healing

**DOI:** 10.1186/s12951-024-02637-8

**Published:** 2024-07-01

**Authors:** Xinyu Zhao, Zenghong Chen, Shuo Zhang, Zhiyuan Hu, Jie Shan, Min Wang, Xu-Lin Chen, Xianwen Wang

**Affiliations:** 1 Department of Burns, The First Affiliated Hospital of Anhui Medical University, Hefei, 230022 P. R. China; 2 Department of Plastic and Reconstructive Surgery, The Second Affiliated Hospital of Anhui Medical University, Hefei, 230601 P. R. China; 3 School of Biomedical Engineering, Anhui Medical University, Hefei, 230032 P. R. China

**Keywords:** Metal-Organic frameworks, Bacterial infection, Wound Healing, Antibacterial agents

## Abstract

Metal-organic frameworks (MOFs) are metal-organic skeleton compounds composed of self-assembled metal ions or clusters and organic ligands. MOF materials often have porous structures, high specific surface areas, uniform and adjustable pores, high surface activity and easy modification and have a wide range of prospects for application. MOFs have been widely used. In recent years, with the continuous expansion of MOF materials, they have also achieved remarkable results in the field of antimicrobial agents. In this review, the structural composition and synthetic modification of MOF materials are introduced in detail, and the antimicrobial mechanisms and applications of these materials in the healing of infected wounds are described. Moreover, the opportunities and challenges encountered in the development of MOF materials are presented, and we expect that additional MOF materials with high biosafety and efficient antimicrobial capacity will be developed in the future.

## Highlights

The structural composition and morphological characteristics of the MOFs are described.

The synthesis methods of MOF materials are described, and the effects of postmodification methods on MOF materials are described;

The antimicrobial mechanism and classification of MOF materials and the antimicrobial characteristics of Ag-, Cu-, and Zn-based MOF materials are described.

This paper highlights the challenges in the development of MOF materials and provides an outlook on the future of MOF materials.

## Introduction

Metal-organic frameworks (MOFs) are a type of metal-organic skeleton compound that consists of self-assembled metal ions or clusters and organic ligands and are crystalline materials with porous mesh structures [1, 2]. According to the literature, MOFs were developed by Kinoshita and Matsubara as early as 1959 [3], and in the following decades, MOFs received less attention. Since the 1990s, when MOFs composed of an infinite polymer with a three-dimensional connected molecular rod framework were synthesized [4], the study of MOFs has received widespread attention, and reports on MOFs have increased annually. In recent years, more than 10,000 studies on MOFs have been reported on the Web of Science every year. MOFs have been widely used in many fields, and in recent years, with the continuous expansion of MOF materials, remarkable results have been achieved in the field of antimicrobial agents [5, 6]. Wound healing is an urgent scientific research problem at this stage and can be categorized into chronic and acute wounds, burn wounds and diabetic wounds, and infected wounds and noninfected wounds according to different classification criteria. At this stage, there are many published reviews related to MOF materials and wound healing, but their research fields are quite different [7–9]. This review focuses on the application of MOF materials in infected wounds.

Infected wounds represent a major challenge in the field of wound healing and can lead to delayed wound healing, increased morbidity and potential complications [10]. Wound infections can be caused by a variety of factors, including surgical procedures, trauma, or underlying medical conditions. The presence of microorganisms (e.g., bacteria, fungi, or viruses) in the wound can disrupt the delicate balance of the wound microenvironment, impede the healing process and contribute to chronic wound formation. The etiology and prevalence of wound infections, as well as the impact of infected wounds on acute and chronic wound healing, have been extensively investigated in the current phase of research [11]. Wound healing is a complex biological process that usually includes hemostasis and clotting, inflammation, blood vessel and granulation tissue formation, and reconstruction and scarring [12, 13]. These stages often intersect and overlap with each other and are affected by multiple factors [14]. Bacterial infection can interfere with all stages of the healing process and delay the healing rate of wounds. Wound infection often leads to increased inflammation, blocked granulation tissue formation, inhibited fibrosis and spread of infection [15]. After bacterial infection of the wound, the release of white blood cells and inflammatory mediators first increases, resulting in a sustained inflammatory response and aggravated tissue damage. Bacterial invasion can also affect cell proliferation, interfere with the formation of blood vessels and granulation tissue in wounds, and delay wound filling. Bacteria also aggravate scar formation by affecting the process of collagen formation and fibrosis in wounds. In addition, bacterial infection of the wound increases the risk of infection spread and can even lead to life-threatening sepsis. The current study showed that the formation of biofilms and microbial communities encapsulated in a protective matrix further exacerbates wound infections and increases the difficulty of treatment [16, 17]. Therefore, timely antibacterial treatment and removal of pathogens are the keys to promoting the healing of infected wounds.

The current phase of research highlights the importance of early detection and appropriate management of infected wounds to optimize healing outcomes. Accurate diagnosis and characterization of causative pathogens by microbial culture or molecular techniques are essential for guiding targeted antimicrobial therapy [18]. The selection of antimicrobial agents should be based on the susceptibility profile of the identified pathogen, taking into account local resistance patterns and individual patient factors [19]. In addition to systemic antimicrobial therapy, local wound management plays a crucial role in the treatment of infected wounds [20]. Various strategies exist to promote wound healing in the presence of infection, including the use of antimicrobial dressings [21], topical antimicrobial agents [22], and advanced wound care modalities such as negative pressure wound therapy [23]. The selection of appropriate wound dressings with antimicrobial properties can help create an environment conducive to healing while reducing the microbial burden [24]. In addition, the emerging field of bioactive materials and biomaterial-based therapies offers promising prospects for the management of infected wounds. The incorporation of antimicrobial peptides [25], growth factors [26], and nanoparticles [27] into dressings or scaffolds to combat wound infections and promote healing has shown promising efficacy. These innovative approaches aim not only to eradicate pathogens but also to promote tissue regeneration and restore the natural wound healing process. Despite advances in the understanding and management of infected wounds, challenges remain. The increase in antibiotic resistance is a serious concern, and alternative treatment strategies need to be explored. Novel antimicrobial agents, including plant-derived compounds, nanoparticles, and photodynamic therapy agents, are being actively investigated at this stage to overcome antibiotic resistance and improve wound healing [28, 29]. In addition, in recent years, strategies to modulate the host immune response [30] and promote an optimal wound healing microenvironment [31] have been explored to address the impact of infected wounds on the healing process.

In summary, infectious wounds significantly impact wound healing outcomes, leading to delayed healing and increased morbidity. Early diagnosis, appropriate antimicrobial therapy, the development of bioactive materials, and advanced therapies provide vast prospects for the treatment of infectious wounds. Sustained interdisciplinary research and the translation of scientific discoveries into clinical practice will further advance the understanding and management of infectious wounds, ultimately improving patient prognosis. In this review, the structural composition and synthetic modifications of MOF materials are systematically introduced, and the antimicrobial mechanisms and applications of these materials in the healing of infected wounds are described. Moreover, several prospects are proposed in light of the opportunities and challenges currently encountered in the development of MOF materials. This review provides a reference for the development of MOF materials and the treatment of infected wounds.

## Structural design of the MOFs

### Composition of the MOFs

**Fig. 1:**
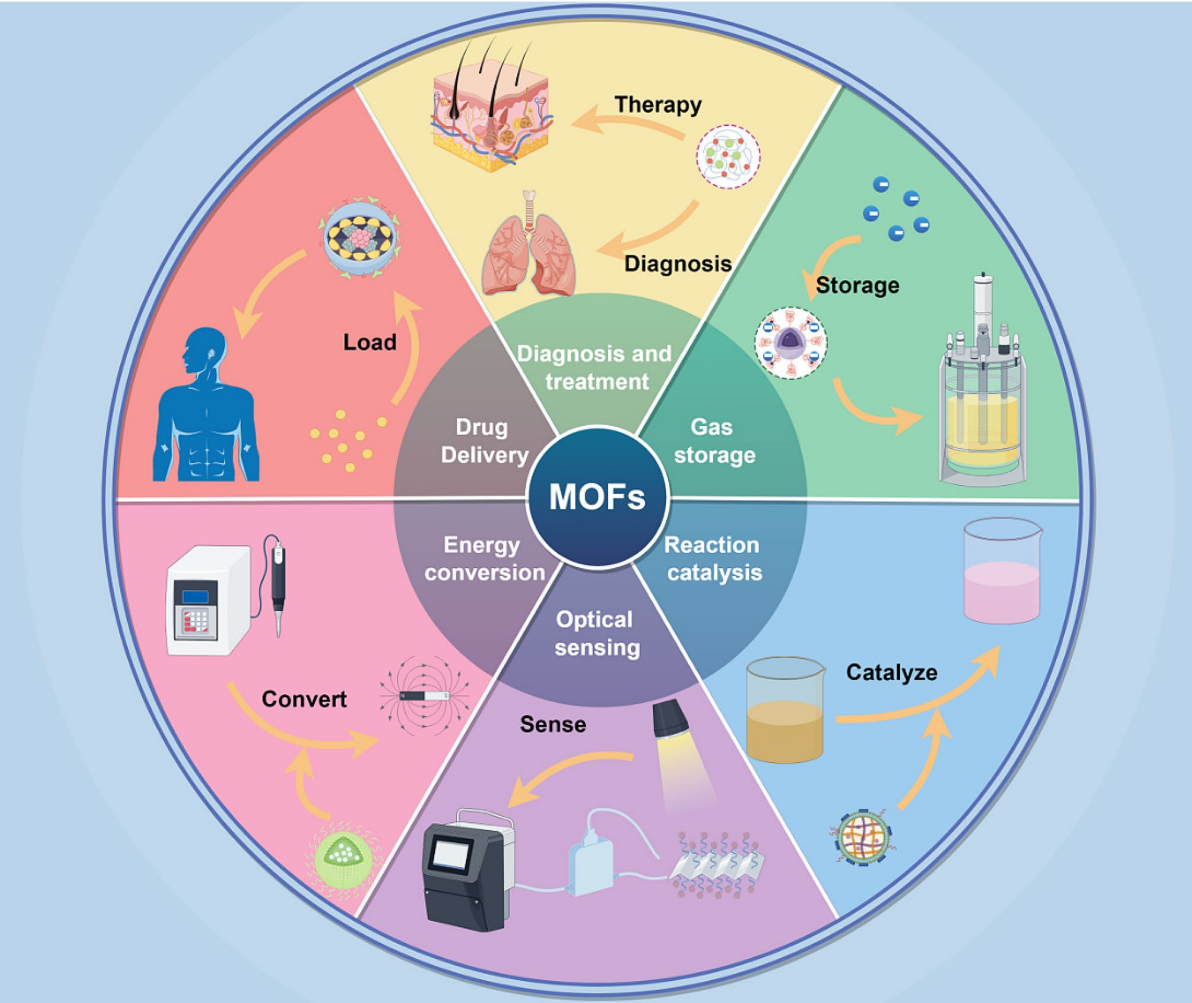
Wide range of applications of MOFs in various fields

MOFs are assembled from metal clusters or metal secondary building units (SBUs) and organic ligands. They are also known as porous coordination polymers [32, 33]. MOFs are mostly formed with metal centers as nodes and organic ligands as linkage bridges. During the coordination process, the diversity of metal sites, organic ligands and coordination modes, as well as the abundance of preparation techniques [34, 35], have resulted in a wide range of MOFs with diverse structures. Silver [36], zinc [37], and copper [38] are common metallic elements in MOFs. Organic ligands often include carboxylic acids, phosphonic acids, nitrogen-containing ligands, etc. MOFs are often characterized by a porous structure and high specific surface area [39] and have a uniform and adjustable pores [40], high surface activity and easy modification [41]; additionally, these materials can be used in a wide range of applications [42–44]. MOFs have been widely used in food preservation [45], gas storage [46], drug delivery [47], optical sensing [48], energy conversion [49], reaction catalysis [50], disease diagnosis and treatment [51], and other fields (Fig. 1). In recent years, with the continuous expansion of MOF materials, remarkable results have been achieved in the field of antibacterial activity.

### Synthesis and postmodification of MOFs

**Fig. 2:**
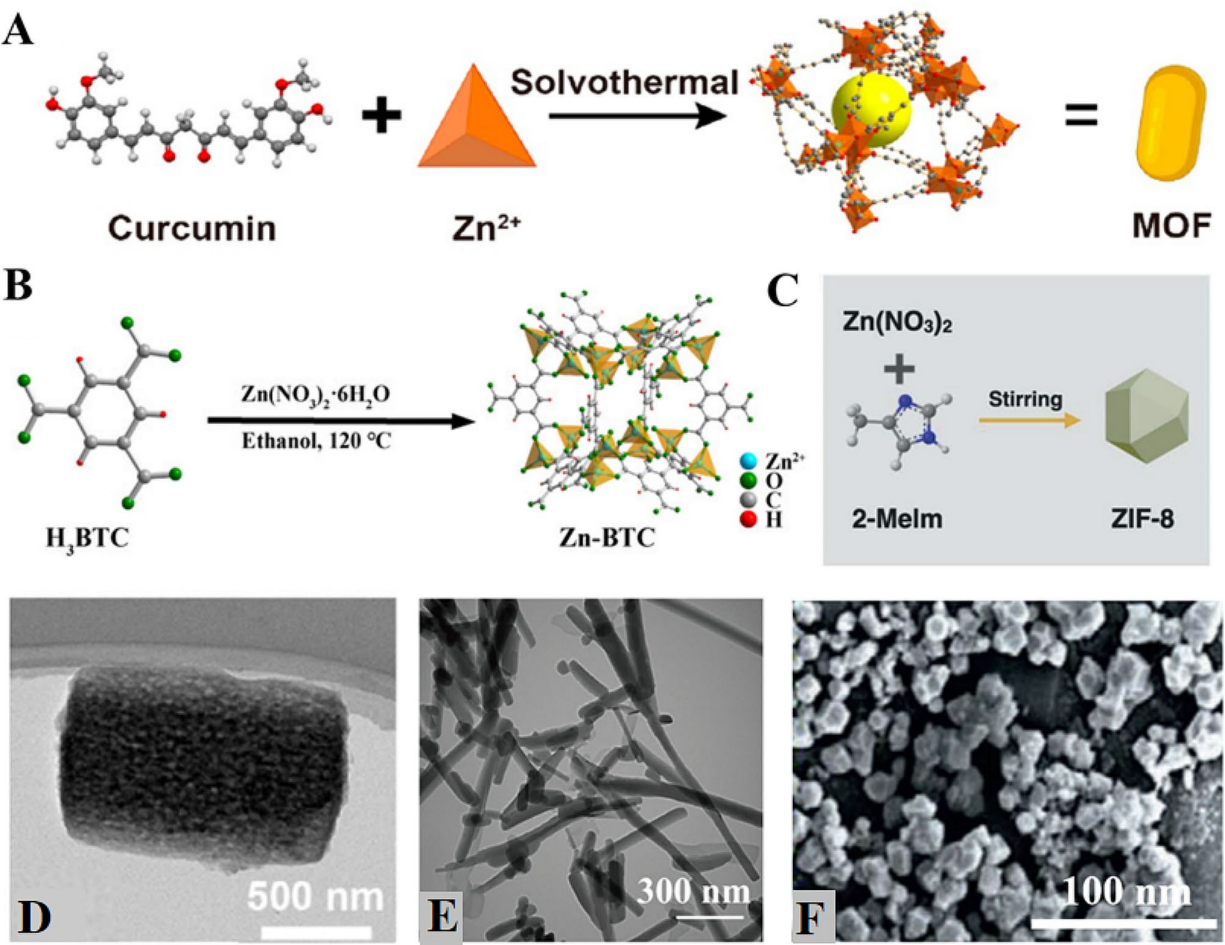
MOFs formed by different organic ligands and Zn^2+^. **A-C**: Schematic diagram of curcumin and **(A)** Zn-Cur, **(B)** Zn-BTC, **(C)** ZIF-8 synthesis process. **D-E**: TEM images of Zn-Cur, Zn-BTC. **F**: SEM images of ZIF-8. Adapted with permission from ref [102]. , Copyright Photothermal hydrogel encapsulating intelligently bacteria-capturing bio-MOF for infectious wound healing. ***ACS nano***, 2022, 16:19491–19,508 [78]. Copyright Zinc-based metal organic framework with antibacterial and anti-inflammatory properties for promoting wound healing. ***Regenerative biomaterials***, 2022, 9: rbac019 [113]. Copyright Zn-MOF encapsulated antibacterial and degradable microneedles array for promoting wound healing. ***Advanced healthcare materials***, 2021, 10:2100056

There are many factors affecting the synthesis of MOFs, among which the selection of metal clusters or SBUs and organic ligands determines the final morphology and function. And diversification of the topology of MOFs can be achieved by changing the metal ions and organic ligands (Fig. 2) [34]. In addition, the solvent, template reagent, reaction pressure, reaction pH, synthesis temperature, and synthesis time also affect the parameters of the MOF, which provides the basis for its diversification and functionalization. To precisely regulate the parameters of the target products and stabilize the synthesis of MOFs with specific morphologies and porosities, various systematic preparation methods have been developed. These methods include hydrothermal/solvothermal methods [52], microwave methods [53], electrochemical methods [54], mechanical methods [55], ultrasonic methods [56], and liquid-phase diffusion methods [57] (Table 1).

**Table 1:** Main preparation methods for MOFs at this stage

Synthetic method	Synthetic path	Synthetic characteristics	Ref.
hydrothermal/solvothermal method	Mix metal salts with organic ligands in proportion with water or organic matter as the solvent.	MOFs are small in size and uniform in structure, with lighter particle agglomeration with higher thermal stability.	[52]
Microwave method	The electromagnetic wave interacts with the moving charge contained in the material, and the heat required by the reaction directly produced by the reactants can realize the rapid crystallization of the ligand.	The reaction was efficient and rapid, and the prepared MOFs had high purity, small particle size and controllable morphology.	[53]
Electrochemical process	Metal electrodes replace metal salts, including anodal dissolution and cathodic deposition. Metal ions are released from the electrode by applying an electric potential and then reacted with the organic ligand in solution.	It reduces the production of corrosive anions, fast preparation process and high porosity of MOFs.	[54]
Mechanical method	To break the precursor internal molecular bond by mechanical grinding and promote the formation of MOFs.	Accelerate the reaction rate and improve the crystallinity of the product.	[55]
Ultrasonic method	Ultrasonic oscillation increases the temperature and pressure of the reaction system, causes the material transfer and chemical reaction in the reaction system, and promotes the formation of MOFs.	The preparation process is simple, with few byproducts and less input cost, but the reaction rate is generally uncontrollable, and the production capacity is low.	[56]
Liquid phase diffusion method	The two insoluble solvents slowly diffuse with each other and finally precipitate crystals.	Simple operation, mild conditions, the need to accurately control the concentration of substances and diffusion rate.	[57]

After the synthesis of MOFs, postsynthesis modification (PSM) of their structures and compositions can modulate their properties and enhance their specific properties [58]. The presence of organic ligands in MOFs makes it easier for them to introduce new functional sites. The modes of postsynthetic modification of MOFs mainly include organic functional group modification [59], metal ion doping [60], biomolecular modification [61], carrier modification [62], surface and structural modification [63], and material functional integration [64]. These postmodification methods can be selected according to the specific application needs of MOFs, and through appropriate postmodification, MOFs can exhibit superior performance in catalysis, drug delivery, bioimaging, diagnosis and therapy.

In brief, the combination of multiple metal elements and organic ligands expands the range of available MOF materials and increases their diversity. The multiple synthesis methods developed at this stage ensure the accurate and efficient preparation of MOF materials, while PSM methods realize the accurate regulation of their properties and functions, which enriches the properties of MOF materials and promotes their development and application.

### Applications of MOFs in the healing of infected wounds

There are several problems with the clinical application of antibiotics. The antibacterial spectrum of a single antibiotic is relatively narrow. Small doses of antibiotics do not completely eradicate pathogens, leading to the persistence and recurrence of infection. High doses of antibiotics often lead to liver and kidney toxicity. Antibiotics are easily removed from the blood circulation, and the special membrane structure of bacteria leads to low penetration of antibiotics. In addition, with the high prevalence of clinical infectious diseases and the extensive use of broad-spectrum antibiotics, the emergence of a large number of superresistant bacteria has led to a lack of effective antimicrobial regimens in the clinic [65, 66]. The development of nanomedicine provides new ideas for solving the problem of bacterial infections. Moreover, nanomaterials can directly disrupt the cell membrane of bacteria by physical action to kill the bacteria, or they can exert enzyme-like activity to produce chemical antibacterially active substances to damage the bacteria [67, 68]. As nanomaterials with special framework structures, MOF materials have good therapeutic efficacy and wide application prospects in the field of antibacterial infections [69–71]. The unique physicochemical properties, morphological structure and catalytic activity of MOF materials determine their wide application in the antibacterial field [72].

### Antibacterial-type MOFs

Currently, antimicrobial MOF materials mainly include self-antibacterial MOF materials [73], MOF materials loaded with antimicrobial nanomaterials or drugs [74], and composite MOF antimicrobial materials [75, 76]. MOFs are tunable complexes that can achieve antimicrobial effects by selecting coordinating metal ions and organic ligands. The metal-activated centers of MOFs have a similar function to that of metal nanoparticles. A large number of metal elements with antimicrobial activity, such as Ag [76], Cu [77], and Zn [78], possess excellent antimicrobial abilities themselves, and after forming MOFs with organic ligands, they can continuously release metal ions and exert antimicrobial effects. Moreover, organic ligands such as porphyrin derivatives and imidazolium salts also have excellent antimicrobial ability, and the MOFs formed by coordination with metal salts can also play direct antimicrobial roles. MOFs have a tunable morphology, and their high specific surface area and porosity provide a basis for loading antimicrobial nanoparticles and drugs [79]. The encapsulation and release of antimicrobial agents can be achieved through postsynthesis modification to play an antimicrobial role. MOFs have antimicrobial properties. MOFs can also be combined with biomaterials such as hydrogels [80], textile fibers [75], and microneedles (MN) [81] to form composite antimicrobial materials, which can jointly achieve antimicrobial properties.

### Antibacterial mechanism

**Fig. 3:**
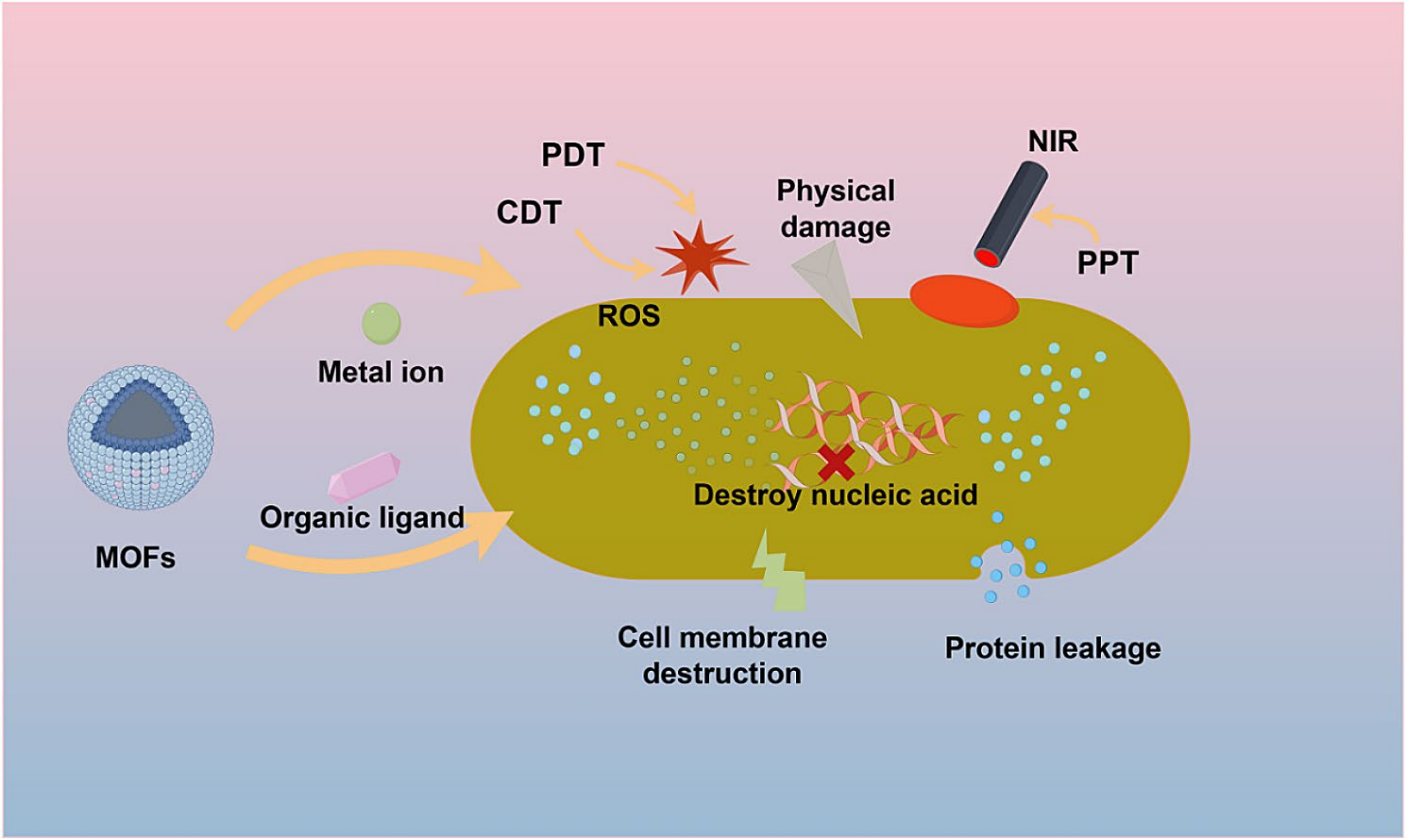
Schematic of the antimicrobial mechanism of MOF materials

The excellent antimicrobial performance of MOFs originates from their reliable antimicrobial mechanism, which is mainly reflected in the high efficiency of antimicrobial action, broad spectrum, stability, and synergistic antimicrobial action of multiple antimicrobial effects without easily causing drug resistance. The antimicrobial mechanism of MOFs is described below (Fig. 3).

#### Classification based on antibacterial dynamics

The antimicrobial activity of MOFs can be classified into two categories according to their dynamic composition: endogenous and exogenous antimicrobial. Endogenous antimicrobial MOFs derive their antimicrobial ability from the metal centers and organic ligands of the MOFs, and their own unique morphology, structure, and catalytic activity are the keys to endogenous antimicrobial activity. Chemodynamic therapy (CDT) is the main method for accessing endogenous antibacterial agents in MOF materials. Exogenous antimicrobial MOFs use light and other external means to stimulate them to produce reactive oxygen species (ROS) or heat sterile [82]; these methods include photodynamic therapy (PDT) and photothermal therapy (PTT). Oxidative stress is an important factor for the antimicrobial activity of MOF materials. The production of antimicrobial active substances relies mainly on the chemodynamic or photodynamic activity of the MOF material itself. Chemodynamic activity refers to the decomposition of metal ions or organic ligands after decomposition to kill bacteria by exerting peroxidase (POD) activity and decomposing H_2_O_2_ to generate ROS [83]. The efficient generation of ROS effectively prevents the cytotoxicity that may be caused by the release of large amounts of metal ions. Photodynamic activity is a therapy in which photosensitizers are activated by visible light to produce ROS and kill bacteria. Porphyrins, phthalocyanines, and porphyrins are commonly used organic ligands with photosensitizing effects [84] that kill bacteria by coupling basal oxygen to generate single linear oxygen. On the other hand, photothermal antimicrobial agents rely on metal or organic ligands with photothermal properties as MOF materials to kill bacteria by generating localized heat treatment under near-infrared (NIR) laser irradiation [85]. This antimicrobial strategy has high therapeutic efficacy and few side effects [86]. Due to the special porous structure of MOF materials, exogenous antibacterial effects can also be achieved by using MOFs as carrier frameworks loaded with materials with photosensitizing or photothermal effects.

#### Classification according to the material structure

MOF materials are classified into four main categories according to their structural characteristics: metal-centered antimicrobial agents, organic ligand antimicrobial agents, carrier drug antimicrobial agents, and composite antimicrobial agents. One of the most important characteristics of MOF materials is the antibacterial activity of metal ions and organic ligands [87, 88].

##### Metal-centered antimicrobial agents

MOF materials connect metal centers with organic ligands via coordination bonds, and the reversible breakage of these ligand bonds causes the metal centers to gradually dissociate to release positively charged metal ions. The surface of bacteria is negatively charged, and dissociated metal ions are often attracted to bacteria due to charge interactions. Metal ions can damage the bacterial cell membrane, penetrate the lipid layer into the bacteria, inactivate and denature important intracellular enzymes and proteins, and ultimately lead to bacterial death [89]. Some specific metal ions often play unique roles in antimicrobial processes. For example, Ag^+^ can interfere with microbial cell membranes, increase membrane permeability, interfere with bacterial respiratory processes, and affect energy production; Ag^+^ can also bind to microbial nucleic acids, preventing bacterial reproduction [90]. Zn^2+^ can disrupt the integrity of bacterial cell membranes and inhibit protein synthesis. Fe^2+^ can cause oxidative stress within bacteria, disrupting microbial cellular structure and metabolism. Fe^2+^ and Cu^2+^ can also exert POD-like enzyme activity, generate ROS, and damage proteins and nucleic acids [91, 92]. Some MOF materials with special morphologies can also play a role in disrupting bacterial cell membranes through physical damage.

##### Antimicrobial effects of organic ligands in MOFs

Organic ligands in MOFs can also be released through the breakage of ligand bonds, and organic ligands such as carboxylic acids [93], porphyrins [94], imidazoles [95], and phenols [96]can remove pathogenic microorganisms through their own antimicrobial effects. Generally, these MOF materials are characterized by rapid antimicrobial activity, broad spectrum, and high biocompatibility. Some organic ligands can also be antimicrobial by PDT or PTT [97, 98].

##### Antimicrobial-loaded MOFs

The porous organic framework of MOFs provides a structural basis for the loading of antimicrobial nanoparticles and drugs. The loaded ions or drugs are released from the pores of MOFs during degradation and play an antibacterial role according to the characteristics of the loaded material. These effects include the chemodynamic effects of nanoparticles and the direct killing effects of drugs that disrupt the bacterial cell membrane and damage intracellular active substances [99].

##### MOF composites for antibacterial treatment

A single antibacterial treatment usually requires a high concentration of antimicrobial agent, and any single antibacterial treatment may result in bacterial inefficiency and the possibility of infection recurrence. The construction of MOF-based composites provides new opportunities to combat bacterial infections. The development of materials such as MOF-composite textile fibers [100], MOF-composite MN patches [101], and MOF-composite antimicrobial hydrogels [102] has led to the simultaneous activation of multiple antimicrobial mechanisms, which can greatly improve antimicrobial efficiency and shorten antimicrobial time.

In summary, MOFs have good antimicrobial properties on their own, and their porous structure can be loaded with efficient antimicrobial particles and drugs. The antimicrobial effect of MOF materials mainly comes from metal ions and organic ligands, and their antimicrobial forms mainly include physical therapy, chemodynamic therapy, photodynamic therapy, photothermal therapy, and antimicrobial agent therapy. Single antimicrobial agents may lead to inefficiency, and MOF composite antimicrobial agents are more advantageous.

### MOF material for treating infected wounds

#### Antimicrobial materials for silver-based MOFs

**Fig. 4:**
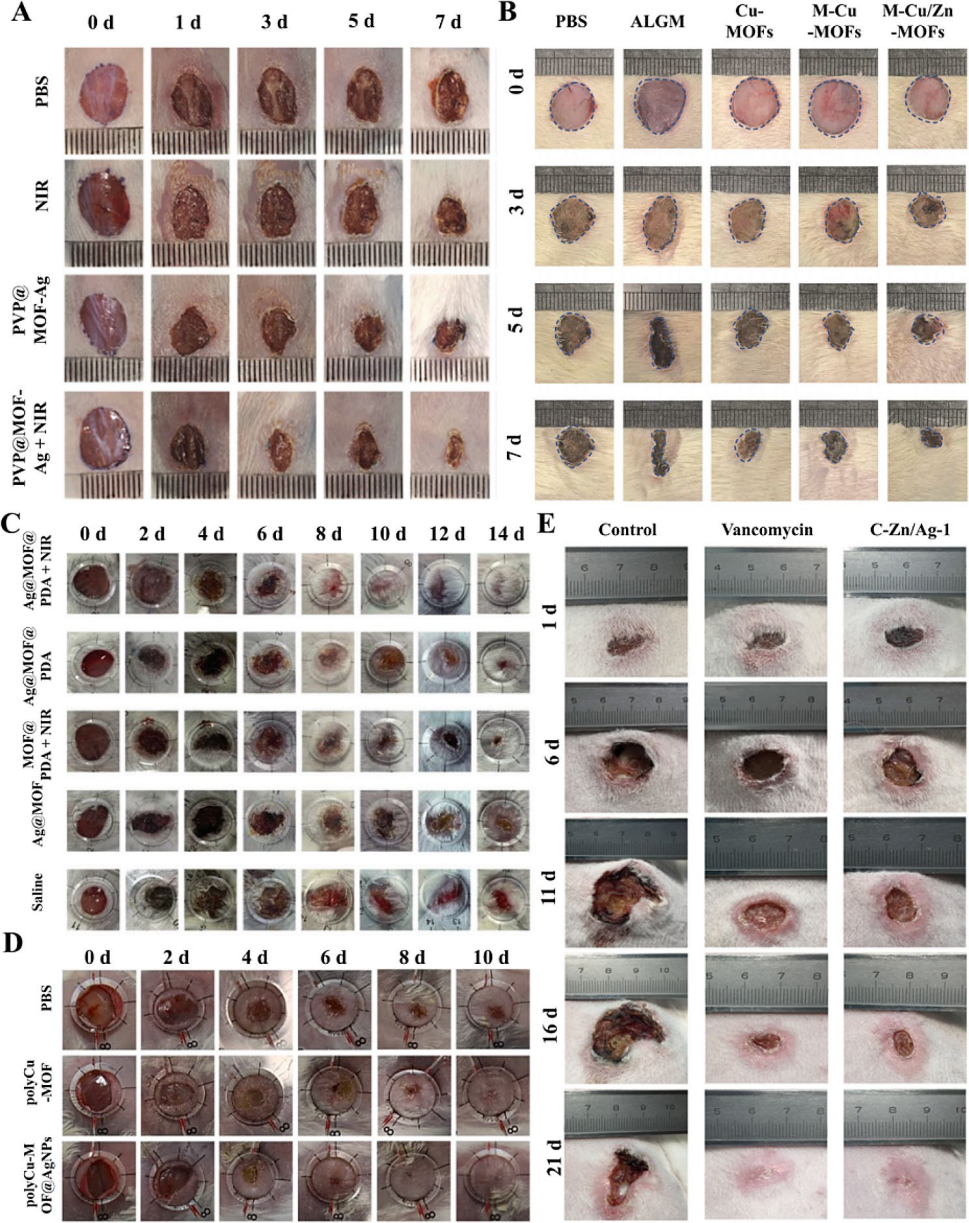
Application of various MOF materials in infected wounds in animals. **A, C, D**: Mouse wound model of *Staphylococcus aureus* infection. **B**: Mouse wound model of *Escherichia coli* infection. **E**: Rabbits infected with *Staphylococcus aureus*. Adapted with permission from ref [119]. , Copyright Near-Infrared Light‐Mediated Cyclodextrin Metal–Organic Frameworks for Synergistic Antibacterial and Anti‐Biofilm Therapies. ***Small***, 2023, 2,300,199 [108]. Copyright metal–organic framework/Ag-based hybrid nanoagents for rapid and synergistic bacterial eradication. ***ACS applied materials & interfaces***, 2020, **12(12)**, 13,698–13,708 [121]. Copyright silver nanoparticle-modified 2D MOF nanosheets for photothermally enhanced silver ion release and antibacterial treatment. ***Acta phys.-chim. Sin***, 2023, **39**, 2,211,043 [112]. Copyright Microfluidic electrospray niacin metal-organic framework-encapsulated microcapsules for wound healing. ***Research***, 2019 [115]. Copyright Copper-based polymer-metal–organic framework embedded with Ag nanoparticles: Long-acting and intelligent antibacterial activity and accelerated wound healing, ***Chemical Engineering Journal***, 2022, **435**, 134,915

In recent years, MOF materials have been used in a wide range of applications for treating infected wounds [24] (Fig. 4). Metal-organic frameworks (Ag-MOFs) constructed on the basis of silver have shown great potential in the antibacterial field due to their high stability, low cytotoxicity, broad antibacterial spectrum, long-lasting antibacterial effect, etc [103]. . Ag^+^ has a strong oxidizing ability; it can disrupt bacterial cell membranes, inactivate intrabacterial proteins, and interfere with the replication of DNA or RNA to produce an efficient and long-lasting antibacterial effect. Liu et al. synthesized three-dimensional Ag-organoboron skeletons with antimicrobial activity [104], and this class of MOF had good killing effects on both gram-positive and gram-negative bacteria. Ag-MOFs synthesized using phosphobenzoic acid as an organic ligand can continuously release Ag^+^ and exhibit excellent antibacterial effects against both gram-positive and gram-negative bacteria [103]. The phosphoric acid and carboxylic acid in phosphobenzoic acid can be coordinated with Ag, and the resulting Ag-MOFs exhibit different morphologies and varying particle sizes. The carboxylic acid group itself has certain antimicrobial activity, and it can form a stable and ordered crystal structure after combining with metal ions. The Ag-MOFs formed after the coordination of carboxylic acid groups with Ag not only maintained excellent antimicrobial ability but also improved the problem of variable morphology [76]. An Ag-MOF (Ag-BTC) synthesized with homotrimellitic acid and imidazole as dual ligands kills bacteria by releasing Ag^+^; on the other hand, the organic ligand homotrimellitic acid can disrupt the structure of the bacteria, imidazole can inhibit DNA synthesis, and the synergistic effect between Ag^+^ and organic ligands enhances antimicrobial performance. Moreover, Ag-BTC has a stable morphology with a homogeneous particle size [105]. Naphthalene diimide (NDI) derivatives with redox activity were synthesized with Ag as a free radical-doped Ag-MOF antibacterial material. It significantly inhibited the growth of *Escherichia coli*, *Pseudomonas aeruginosa*, *Bacillus subtilis* and *Staphylococcus aureus* and significantly accelerated the healing of infected wounds in mice in vivo [106]. Studies have also reported 3D laminated nanofiber sponges containing curcumin (3D-AgMOF-CUR) [107], demonstrating the role of MOFs in inhibiting bacterial growth.

In a nutshell, Ag combined with organic ligands can form stable and homogeneous MOF materials, and the sustained release of Ag + combined with antimicrobial organic ligands can synergistically exert efficient and long-lasting antimicrobial effects. These multiple antimicrobial strategies involving Ag-based MOFs have potential applications in promoting wound healing.

#### Antimicrobial materials for zinc-based MOFs

**Fig. 5:**
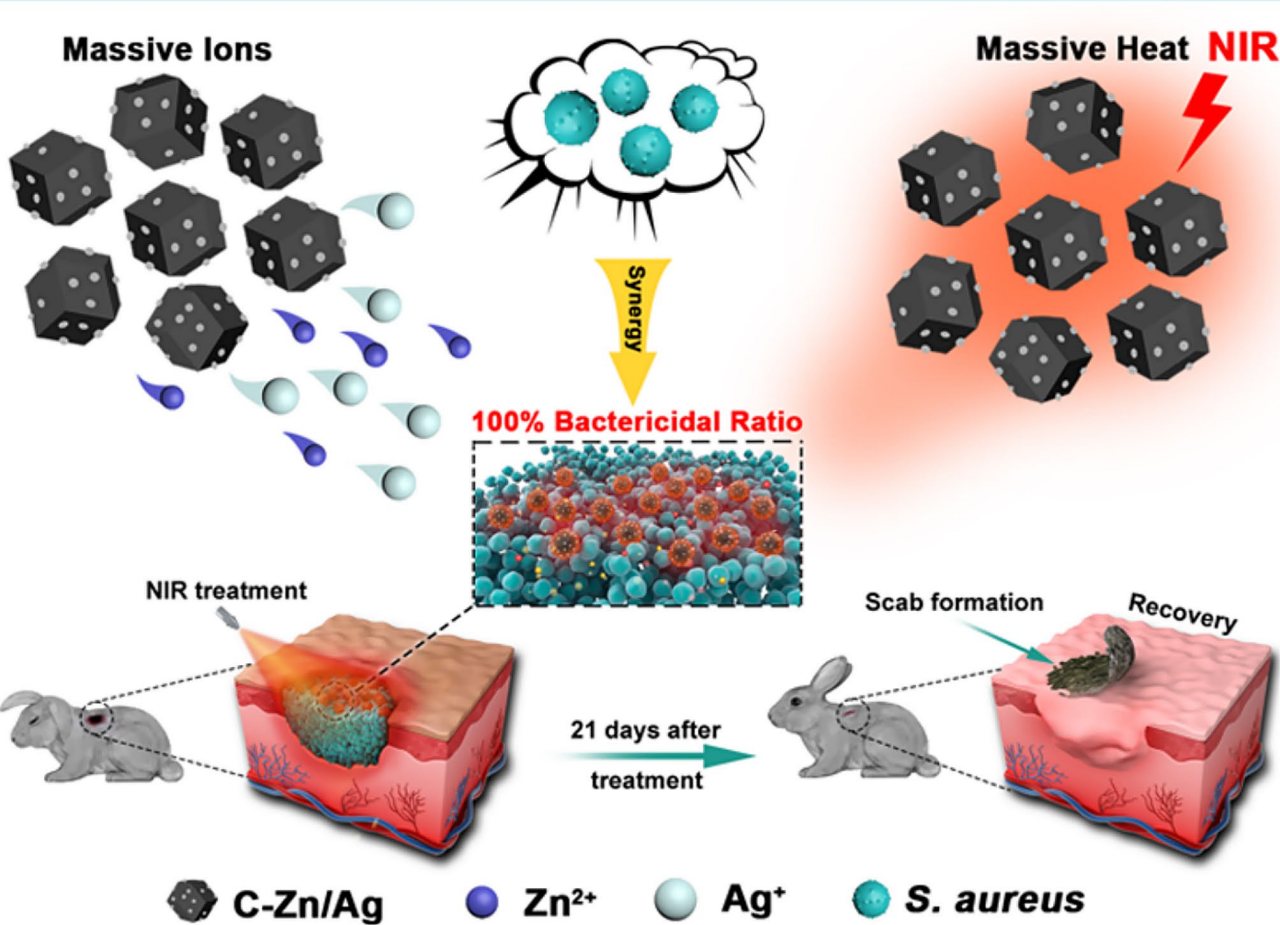
Schematic diagram of Ag-doped Zn-like graphite carbon skeleton derivatives (C-Zn/Ag) efficiently release Zn^2+^ and Ag^+^ ions combined with photothermal conversion ability for synergistic antibacterial. Adapted with permission from ref [108]. , Copyright Metal–organic framework/Ag-based hybrid nanoagents for rapid and synergistic bacterial eradication. ***ACS applied materials & interfaces***, 2020, **12(12)**, 13,698–13,708

Zn is a trace element required by the human body. Zn itself has antimicrobial properties, and the introduction of Zn into MOFs can enhance their stability and biocompatibility. Zn-MOFs can produce antimicrobial effects by releasing Zn ions and organic ligands, or they can exert antimicrobial effects by loading antimicrobial agents. Yang et al. reported a MOF-derived nanocarbon (C-ZIF) consisting of Zn and a graphite-like carbon skeleton and introduced Ag nanoparticles through a substitution reaction between Zn and Ag^+^ to form a Ag-doped MOF derivative (C-Zn/Ag). This MOF material can release Zn^2+^ and Ag^+^ ions efficiently and has a strong photothermal conversion ability to realize synergistic chemodynamic and photothermal antimicrobial effects, allowing complete killing of *Staphylococcus aureus* and *Escherichia coli* at a very low dose (Fig. 5) [108]. Another study used Zn^2+^ and curcumin, equipped with vancomycin (Van) and quaternary ammonium chitosan (QCS). The MOFs combined with a dual-network hydrogel can accurately capture bacteria and achieve rapid killing by releasing Zn ^2+^ and Van [102]. Zeolite imidazolate framework-8 (ZIF-8) is an excellent biocompatible MOF with promising applications in treating bacterial infections. Studies have reported the successful construction of Ag-Phy@ZIF-8 by encapsulating Ag NPs in ZIF-8 combined with physcion (Phy). Ag-Phy@ZIF-8 modified with hyaluronic acid (HA) had potent inhibitory effects on *Escherichia coli* and *Staphylococcus aureus*, as well as good biocompatibility, suggesting that this material could be used in smart wound excipients with potential applications [109]. Wang et al. developed a ZIF-8 material combined with a Zn-doped MoS_2_ hybrid material (ZnDMZ) that exhibited photocatalytic and photothermal effects under 660 nm light irradiation, and the combined release of Zn^2+^ had strong antibacterial effects. In animal experiments, ZnDMZ was able to effectively treat bacterium-infected open wounds [110]. ZIF-8 can decompose under acidic conditions. Song Z et al. developed a photoresponsive antibiotic delivery system based on o-nitrobenzaldehyde (o-NBA)-modified MOFs (o-NBA@ZIF-8). o-NBA possesses photoresponsive properties, and 365 nm UV light irradiation can reduce the pH, promote the degradation of ZIF and the release of loaded drugs, and effectively promote the healing of infected wounds [69]. Ashmawy SR et al. synthesized zinc acetate/nicotinic acid metal-organic frameworks (Zn-NA MOFs) using nicotinic acid (NA) and zinc (Zn) via simple synthesis methods and rapid reaction processes. The Zn-NA MOFs were pH dependent and could sustain the release of NA and Zn in a weakly alkaline environment, which was effective at killing *Staphylococcus aureus*, *Escherichia coli* and *Pseudomonas aeruginosa*. Moreover, in vivo experiments demonstrated that Zn-NA MOFs significantly reduced the wound area and promoted tissue regeneration during wound healing [111]. On the other hand, a MOF (Zn-BTC) based on homobenzoic acid (H_3_BTC) with zinc ions demonstrated excellent antibacterial and anti-inflammatory properties and effectively promoted wound healing. Zn-BTC achieved dual bactericidal and antioxidant effects by releasing zinc ions, disrupting microbial membranes and activating antioxidant enzymes. In vivo experiments confirmed the favorable effect of Zn-BTC on skin wound healing in SD rats [78]. Alginate shell microcapsules encapsulated with niacin (NA)-Cu/Zn MOFs synthesized by the microfluidic electrospray method could kill bacteria by intelligently and controllably releasing calcium, copper and zinc ions according to the degree of infection and disruption of the bacterial biofilm. In addition, the NA-Cu/Zn MOFs activate copper/zinc superoxide dismutase (Cu/Zn-SOD) to eliminate oxygen free radicals. The released nicotinic acid promotes vasodilation and the absorption of functional metal ions. In vivo studies have shown that NA-Cu/Zn MOFs can shorten the healing time of infected wounds [112]. Yao et al. successfully prepared an array of biodegradable methacrylate hyaluronic acid (MeHA) MNs encapsulated with Zn-MOFs. The photocrosslinked degradable MN arrays were able to stabilize the release of Zn^2+^, disrupt the bacterial pod membrane, and exhibit excellent antibacterial activity [113].

Zn-based MOF materials have good loading capacity, can encapsulate all kinds of antibacterial substances, have photocatalytic and photothermal effects to kill bacteria, and ultimately promote wound healing. As one of the most common loading materials, Zn-based MOFs provide an extensive research and development basis for antimicrobial MOFs.

#### Antimicrobial materials for copper-based MOFs

**Fig. 6:**
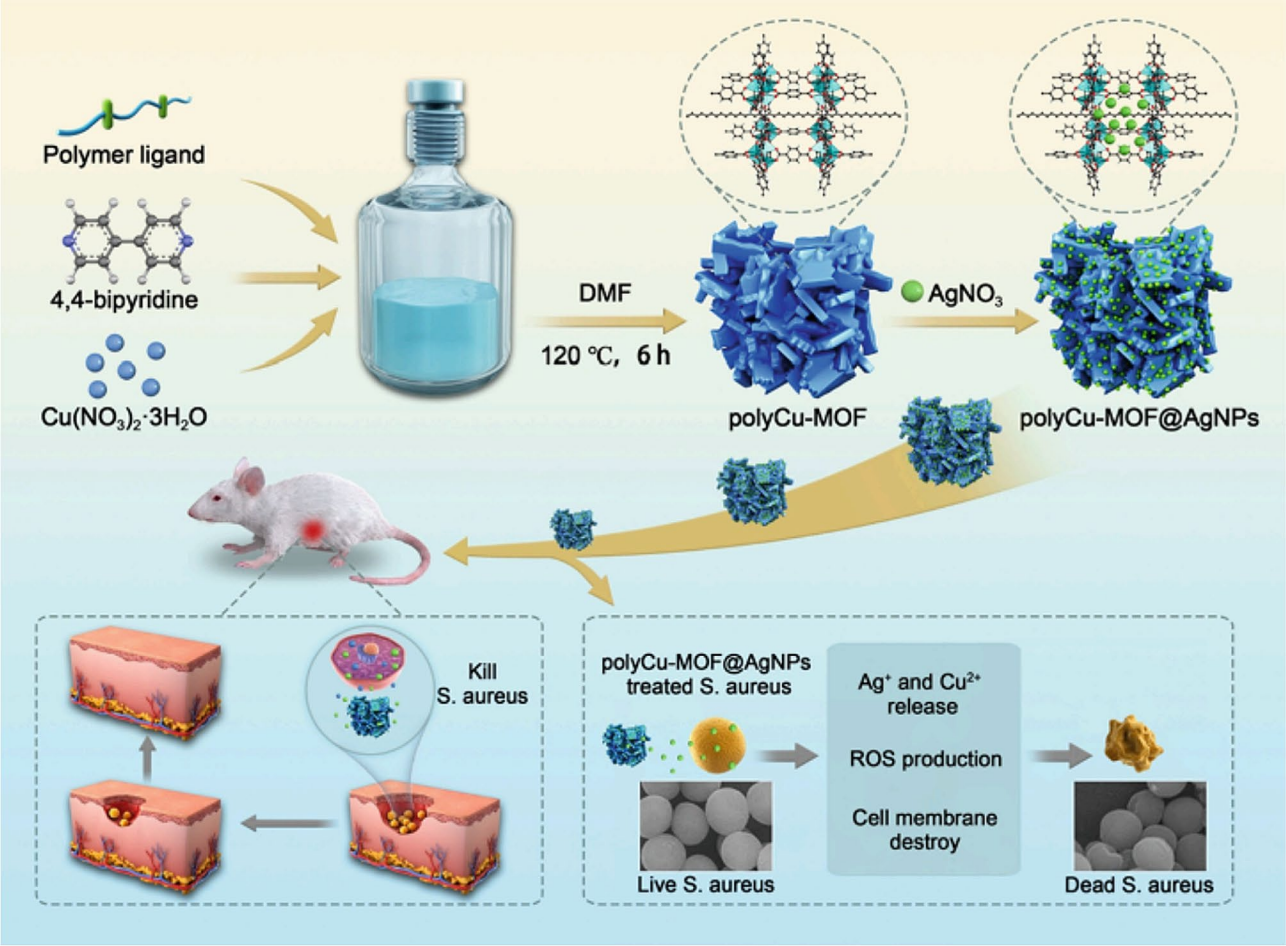
Schematic diagram of the synthesis procedure and antibacterial and wound healing activities of polyCu-MOF@AgNPs hybrid. Adapted with permission from ref [115]. , Copyright Copper-based polymer-metal–organic framework embedded with Ag nanoparticles: Long-acting and intelligent antibacterial activity and accelerated wound healing, ***Chemical Engineering Journal***, 2022, **435**, 134,915

**Fig. 7:**
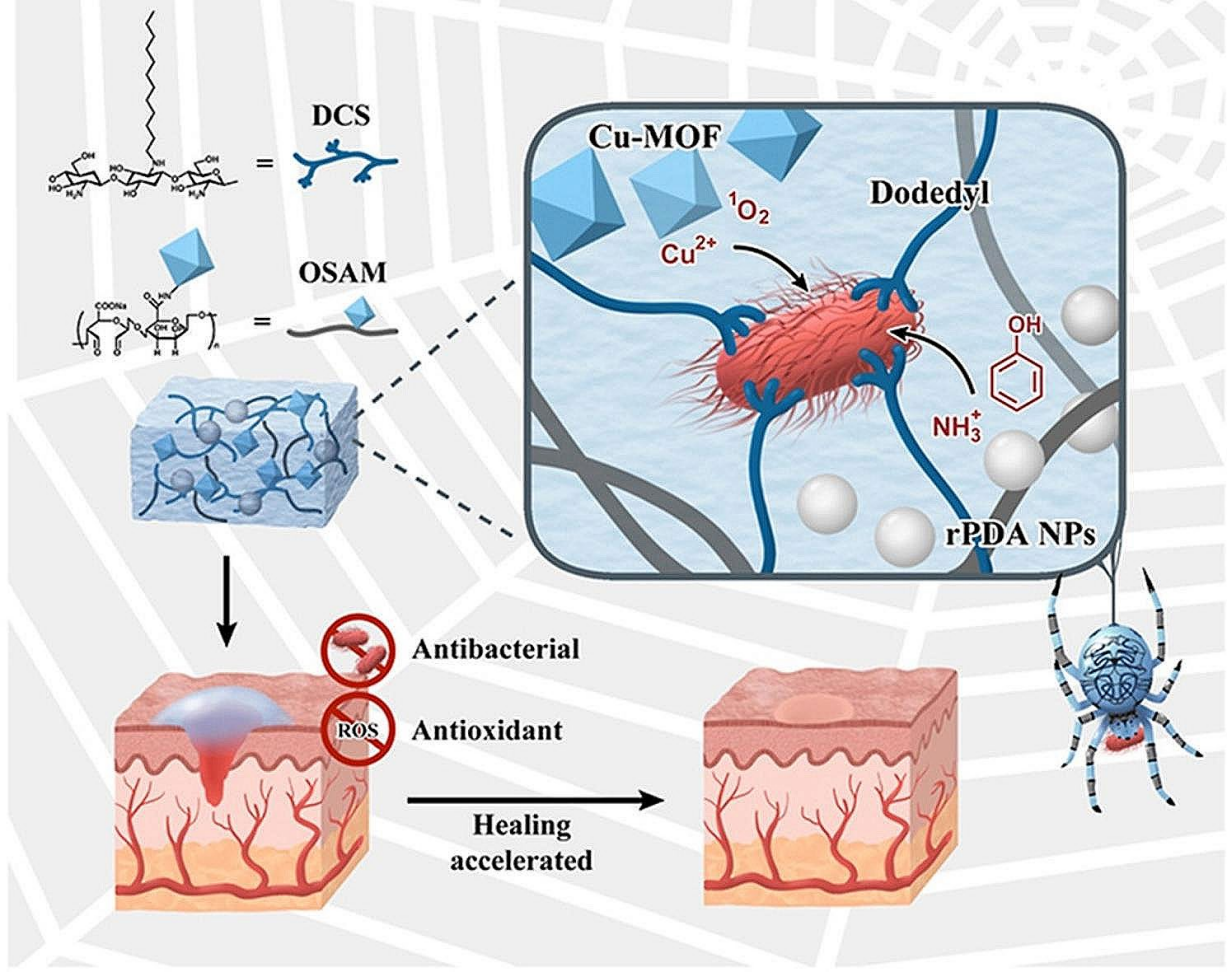
Schematic diagram of the bioinspired synergistic antibacterial hydrogel for synergistic antibacterial and whole-process promotion of wound healing. Adapted with permission from ref [117]. , Copyright rPDAs doped antibacterial MOF-hydrogel: Bioinspired synergistic whole-process wound healing. ***Materials Today, Nano***, 2023, 100,363

Copper is an essential trace element in the human body and has a wide range of antimicrobial applications. A variety of copper-based MOF materials with antimicrobial properties have been developed that kill bacteria by releasing Cu^2+^ and generating ROS. A polyacrylamide (PAM) gel wound dressing (MOF(Fe-Cu)/GOx-PAM) consisting of a copper-iron bimetallic organic framework MOF (Fe-Cu) loaded with glucose oxidase (GOx) was successfully prepared by a molding method. The decomposition of glucose by GOx produces abundant H_2_O_2_, and due to the doping of copper, the catalytic performance of the bimetallic MOF (Fe-Cu) on POD-like enzymes was approximately 5 times greater than that of the monometallic MOF (Fe). The MOF (Fe Cu)/GOx PAM gel also induced M_2_ macrophage polarization, accelerating angiogenesis and neurogenesis. In vivo experiments have also demonstrated that MOF (Fe Cu)/GOx PAM can effectively promote the healing of infected wounds through synergistic antimicrobial and inflammatory modulation [114]. Copper-based polymer-metal-organic frameworks (polyCu-MOFs) were prepared using a polyether ligand of the 1,4-benzenedicarboxylic acid (H_2_BDC) unit, 4,4-bipyridine, coordinated to copper. Ag ions were adsorbed in the polyCu-MOF network and reduced using NaBH_4_ to form polyCu-MOF@AgNP hybrids. polyCu-MOF@AgNPs effectively kill bacteria and promote the healing of infected wounds by affecting bacterial metabolism (Fig. 6) [115]. Mo et al. synthesized a G-quadruplex/heme DNAzyme aptamer (Apt-DNAzyme) and tannic acid-chelated gold nanoparticle (Au-TA)-modified copper-based MOF nanosheets (GATC). GATC was able to increase POD-like activity and produce more hydroxyl radicals (OH) to kill bacteria. The released Apt-DNAzyme was able to recognize and bind to bacteria, increasing the surface contact area of the bacteria. In addition, GATC was able to consume GSH to avoid OH depletion and enhance its bactericidal effect. In vitro experiments have shown that GATC can effectively promote the healing of infected wounds [116]. A reduced polydopamine nanoparticle (rPDA)-doped copper-based metal-organic framework (Cu-MOF)-hydrogel (GEL-MOF-rPDA) was able to effectively promote the healing of infected rat wounds. In this case, dodecyl chitosan-oxidized sodium alginate was constructed as a hydrogel via Schiff base cross-linking, with dodecyl tails used to trap bacteria and sustained release of Cu^2+^ and rPDAs for synergistic antimicrobial activity (Fig. 7) [117]. Spherical Cu-TCPP MOFs were synthesized using 5,10,15,20-tetrakis (4-aminophenyl) porphyrin (TCPP) as an organic ligand coordinated to Cu. Ag-CuTCPP MOFs were obtained by encapsulating Ag nanoparticles, and compared to the original Cu-TCPP MOFs, Ag-CuTCPP MOFs exhibited enhanced antibacterial ability and very low cellular toxicity. In vitro experiments also demonstrated that Ag-CuTCPP MOFs could effectively promote the healing of infected wounds [77]. However, more copper ions tend to cause some cytotoxicity, and the development of Cu-based MOF materials can effectively reduce Cu^2+^ release and cytotoxicity while ensuring antibacterial effects [118].

The Cu^2+^ released by the Cu-based MOF materials has an excellent POD enzyme effect; through the release of OH-, it can effectively kill bacteria and promote the healing of various types of infected wounds. Cu^2+^ often has high cytotoxicity, and the development of Cu-based MOF materials has reduced its cytotoxicity, effectively guaranteeing biosafety.

#### Other antimicrobial MOF materials

**Fig. 8:**
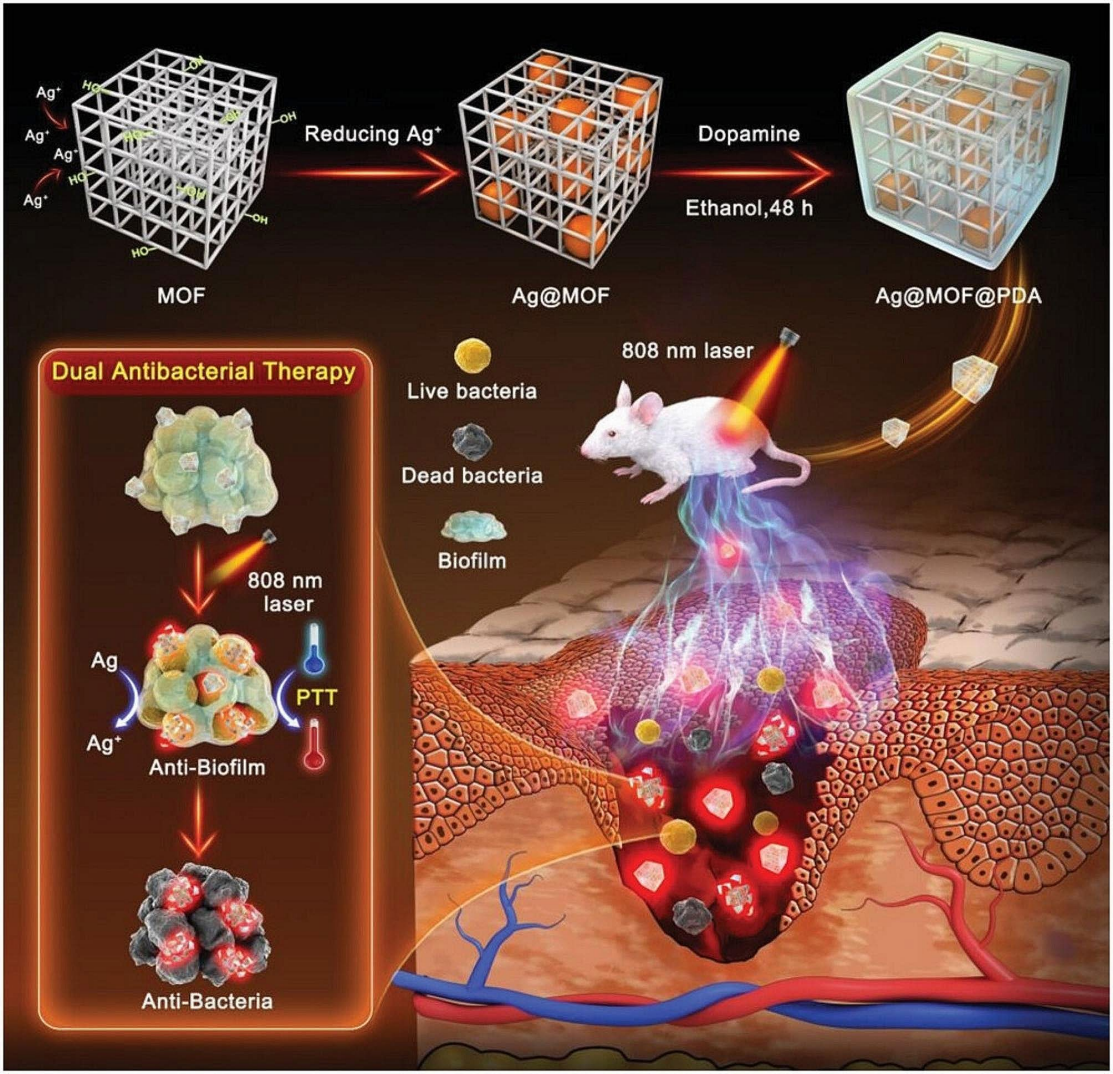
Schematic illustration of the synthetic route of Ag@MOF@PDA and its synergistic antibacterial and antibiofilm effects. Adapted with permission from ref [119]. , Copyright Near-Infrared Light‐Mediated Cyclodextrin Metal–Organic Frameworks for Synergistic Antibacterial and Anti‐Biofilm Therapies. ***Small***, 2023, 2,300,199

**Fig. 9:**
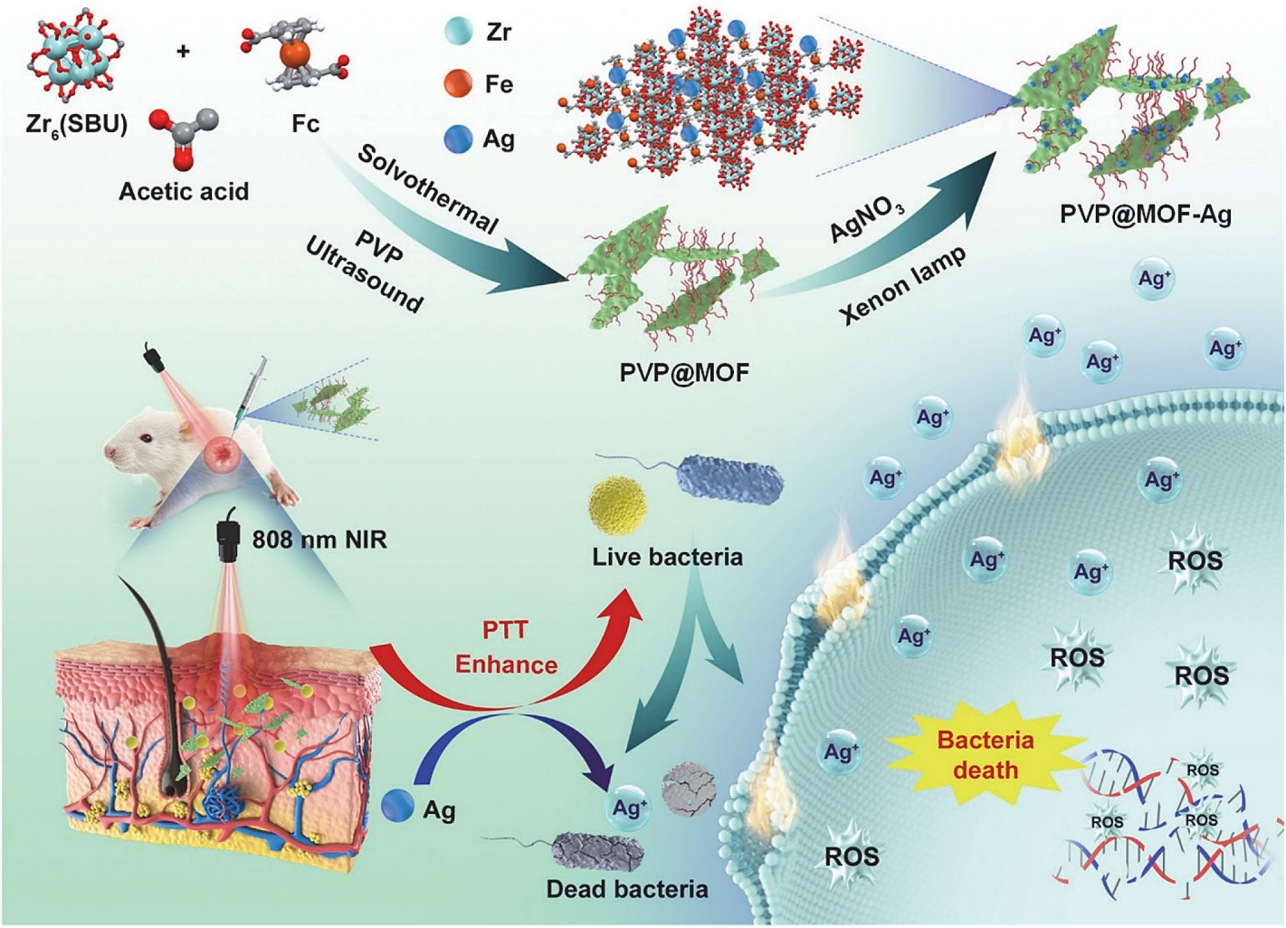
Schematic illustration of the preparation of PVP@MOF-Ag nanosheets and its photothermally enhanced Ag^+^ release for bacterial therapy. Adapted with permission from ref [121]. , Copyright Silver nanoparticle-modified 2D MOF nanosheets for photothermally enhanced silver ion release antibacterial treatment. ***Acta phys.-chim. Sin***, 2023, **39**, 2,211,043

In addition to Ag-MOFs, Zn-MOFs, and Cu-MOFs, which have a wide range of applications in infected wounds, there are many other metal ions that can form MOFs with specific antimicrobial efficacy. The synthesis of ultrafine Ag NPs by cyclodextrin metal-organic skeletons (CD-MOFs) and modification with GRGDS peptides achieved efficient bacterial inhibition and promoted hemostasis in aqueous media. This strategy offers great promise for the design of effective wound healing devices [99]. Another kind of porous silver nanoparticle (AgNP) was synthesized by polymerizing dopamine onto the surface of CD-MOFs. The combination of Ag^+^ release and photothermal therapy can quickly kill bacteria and eradicate biofilms (Fig. 8) [119]. Iron-based metal-organic frameworks (Fe-MOFs) are promising antimicrobial substitutes because they have strong POD enzyme activity and can generate strong ROS against bacterial infections. Iron frameworks are unstable under physiological conditions, and the Fe-MOF (ZFM) nanoenzymes modified with trace Zn developed by Zhong, D et al. can improve the stability of MOFs, reduce the release of large amounts of iron ions, and decrease the toxicity of FMs. ZFMs are still highly lethal against ultrabroad-spectrum β-lactamase-producing *Escherichia coli* and can effectively promote the healing of infected wounds [120]. Studies have also reported 2D MOF nanosheets with photothermally enhanced silver ion-releasing antimicrobial treatment (Fig. 9) [121]. Bismuth (Bi) metal-organic frameworks (MOFs) have been used less frequently. Wu et al. coordinated tetrakis (4-carboxyphenyl) porphyrin (TCPP), an organic ligand with Bi ions, to form Bi-TCPP. Bi-TCPP possesses ultrafast unilinear oxygen-generating capacity and high photothermal conversion efficiency under 660 nm light irradiation and can effectively heal infected wounds [122]. Cu^2+^ was doped into the porphyrin ring of an MOF (PCN 224) composed of zirconium (Zr). This Cu^2+^-MOF material possesses photothermal properties, while doped Cu^2+^ can trap electrons and enhance the photocatalytic performance of the MOF under 660 nm light irradiation. In vivo results showed that Cu^2+^-MOFs could effectively kill bacteria and accelerate wound healing [123]. The modified Zr-based porphyrin MOFs (Au NCs @ PCN) prepared by another in situ growth method have good ROS generation capacity and photothermal effects and can effectively inhibit the activity of drug-resistant bacteria and promote the healing of infection and diabetes [124]. Ultrathin 2D aluminum-based porphyrin MOFs were prepared by ultrasonic stripping. Ultrasmall AuNPs are loaded onto MOFs by in situ reduction (UsAuNPs/MOFs). Au NPs and 2D MOFs synergically exert PoD-like effects and promote the healing of infected wounds [125].

MOF antibacterial materials mainly contain metal elements or organic ligands and have a broad antibacterial spectrum and sustained antibacterial effects. Both endogenous and exogenous methods for exerting antibacterial effects ensure the stability of MOF materials in the treatment of infected wounds (Table 2). At the same time, the development of MOFs greatly reduces the cytotoxicity caused by the release of large amounts of metal ions and ensures the safety of treatment. The MOF materials used to treat infected wounds are mainly Ag, Zn and Cu. The slow and effective release of their metal centers enhances CDT, PDT and PTT, and the combination of special organic ligands results in high efficiency and targeting of the treatment. In recent years, the development of a variety of new composite antimicrobial MOF materials has led to new ideas for the multifunctional treatment of infected wounds.

**Table 2:** The application of MOF materials in infected wounds

Metal ion	Mofs	Ligand	Preparation method	Antibacterial mechanism	Category	Ref.
Ag^+^	Ag-MOF	Tris(4-pyridylduryl)borane	Hydrothermal method	Ag^+^ and ligand	Endogenous	[104]
	Ag-BTC	Homotrimellitic acid	Solvent method	Ag^+^ and ligand	Endogenous	[105]
	Ag-MOF	Naphthalenediimide	Precipitation method	Ag^+^ and ROS	Endogenous	[106]
	2D Ag-MOF	Acetic acid	Hydrothermal method	Ag^+^ and PTT	Endogenous Exogenous	[121]
	Ag-MOF	2-Methylimidazole	Hydrothermal method	Ag^+^ releasing	Endogenous	[107]
Zn^2+^	C-ZIF	Graphite-like carbon skeleton	In situ displacement reaction	Zn^2+^, Ag^+^, PTT and CDT	Endogenous Exogenous	[108]
	ZIF-8	2-methylimidazole	The double solvents method	Zn^2+^, Ag^+^ and ligand	Endogenous	[109]
	Zn-DMZ	N, N dimethylformamide	Ultrasonic solvent stripping method	Zn^2+^ and PDT	Endogenous Exogenous	[110]
	ZIF-8	2-methylimidazole	One-pot method	Zn^2+^ and loaded drugs	Endogenous Exogenous	[69]
	Zn-NA MOF	Nicotinic acid	Precipitation method	Zn^2+^ and ligand	Endogenous	[111]
	Zn-BTC	Homophthalic acid	Hydrothermal method	Zn^2+^ releasing	Endogenous	[78]
	Zn MOF	Nicotinic acid	Microfluidic electrospray method	Zn^2+^, Cu^2+^ releasing	Endogenous	[112]
	ZIF-8	2-methylimidazole	molding method.	Zn^2+^ releasing	Endogenous	[113]
Cu^2+^	MOF(Fe-Cu)	2-Aminobenzoic acid	Hydrothermal method	ROS	Endogenous	[114]
	Cu-MOF	4,4′-bipyridine	Wet chemical method	Cu^2+^ and ROS	Endogenous	[115]
	Cu-MOF	2-Methylimidazole	Precipitation method	ROS	Endogenous	[116]
	Cu-MOF	Oxidized sodium alginate	Hydrothermal method	Cu^2+^ and rPDAs	Endogenous	[117]
	Cu-MOF	5,10,15,20-Tetrakis (4-aminophenyl) porphyrin	Hydrothermal method	Ag^+^, Cu^2+^releasing	Endogenous	[77]
Fe^3+^	Fe-MOF	2-aminoterephthalic acid	Solvothermal methods	ROS	Endogenous	[120]
Bi^3+^	Bi-TCPP	Tetra(4-carboxyphenyl) porphine	One-step hydrothermal method	ROS and PTT	Endogenous Exogenous	[122]
Zr^2+^	PCN-224	Tetra(4-carboxyphenyl) porphine	Hydrothermal method	PDT, CDT and PTT	Endogenous Exogenous	[123]
	PCN-224	Tetra(4-carboxyphenyl) porphine	In situ growth method	PDT and PTT	Endogenous Exogenous	[124]
Al^3+^	Al-MOFs	porphyrin	In situ reduction	ROS	Endogenous	[125]

### Challenges and opportunities

**Fig. 10:**
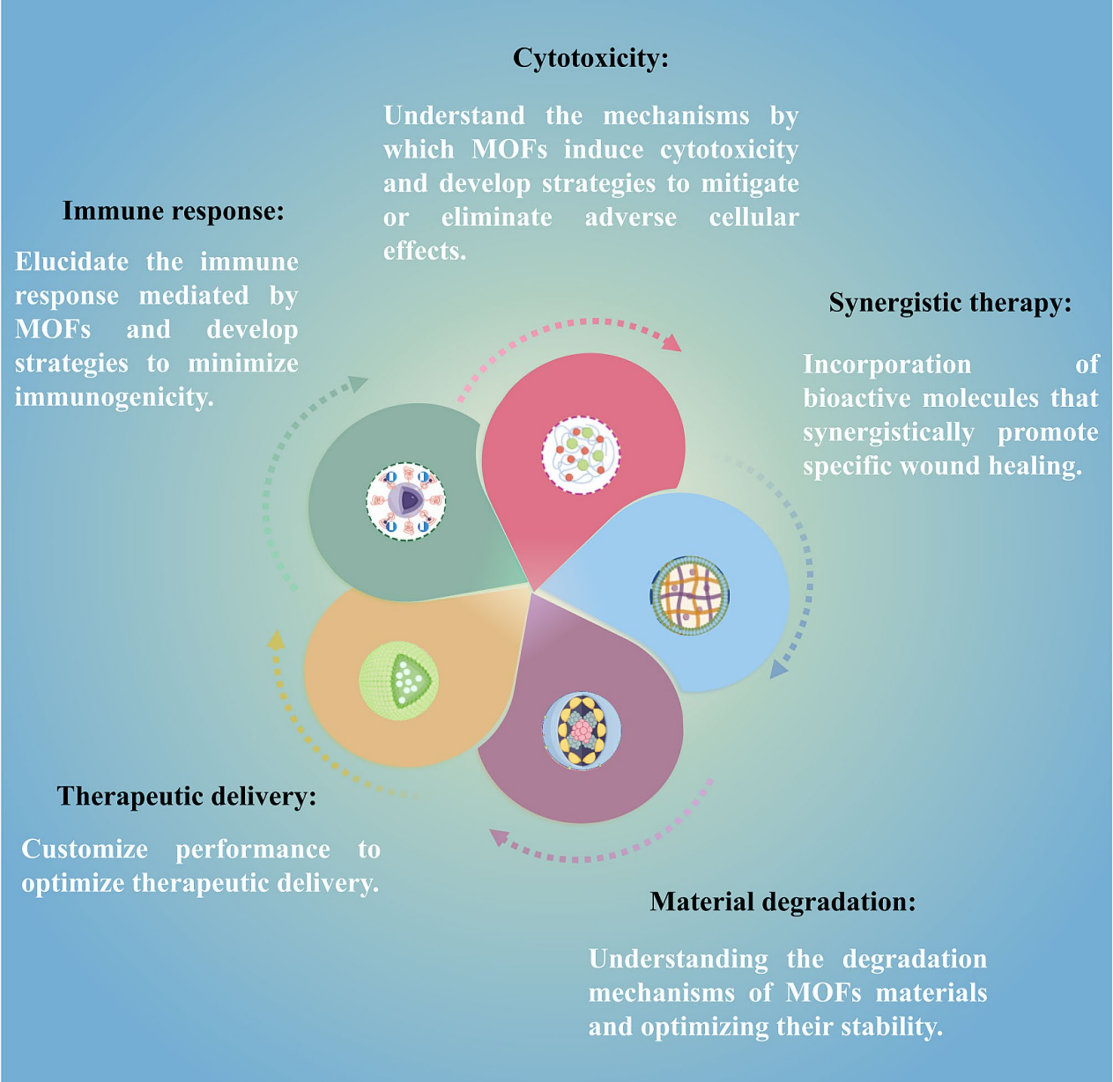
Challenges and opportunities for MOFs

MOFs are promising biomedical materials for a wide range of applications (Fig. 10), but their biocompatibility and safety are key to their successful clinical application. All safety issues affecting the application of MOF materials in vivo should be seriously appreciated. Various in vitro studies have evaluated the effects of MOFs on various cell types, including hepatocytes, fibroblasts, keratinocytes, and immune cells [126]. These studies have shown that MOFs can exhibit varying degrees of cytotoxicity [127, 128], depending on their composition, size, and surface properties. MOFs have been reported to trigger an immune response [129], including the production of proinflammatory cytokines, which may affect wound healing outcomes. In addition, MOFs can degrade over time, releasing their constituent components, and long-term biocompatibility is a key consideration for MOF materials in wound healing. Degradation products may have different biological effects and should be carefully evaluated for potential toxicity or unwanted side effects. Future studies will focus on understanding the mechanisms by which MOFs induce cytotoxicity and developing strategies to mitigate or eliminate any adverse cellular effects. Evaluating the immunogenicity and inflammatory response of MOF materials is essential for assessing their safety in wound healing applications. Future studies will aim to elucidate the factors responsible for MOF-mediated immune responses and develop strategies to minimize immunogenicity, such as surface modification or the use of biocompatible coatings. At the same time, future studies will also focus on understanding the degradation mechanisms of MOF materials, optimizing their stability, and designing MOFs with controlled degradation rates to ensure long-term biocompatibility.

On the other hand, the importance of controlling the pore size, surface charge, and functionalization of MOFs to enable targeted drug release at the wound site will be achieved by fine-tuning the properties, which will lead to the development of MOFs with improved drug loading capabilities. Future research on the use of MOFs as wound-healing materials will focus on tailoring their properties to optimize therapeutic delivery. In addition, the exploration of stimulus-responsive MOFs (such as ROS and PH) has led to promising on-demand drug release methods based on specific wound conditions [130, 131]. Some MOFs encapsulating bioactive molecules have shown enhanced wound healing outcomes. Future studies could explore the synergistic effects of combining multiple growth factors or incorporating other bioactive molecules to promote specific wound healing processes.

In summary, the promise and feasibility of the clinical application of MOF materials in wound healing offer promising avenues for advancing the field. The tunable porosity, high surface area and customizable unique properties and functionalities of MOFs enable tailored therapeutic interventions and enhanced wound healing interactions. Future research efforts will include customizing MOF properties, integrating bioactive molecules and growth factors, designing smart dressings, expanding wound bioengineering applications, and addressing biocompatibility issues.

## Data Availability

No datasets were generated or analysed during the current study.
